# Efficacy and Safety of Repeated Courses of rTMS Treatment in Patients with Chronic Subjective Tinnitus

**DOI:** 10.1155/2015/975808

**Published:** 2015-10-25

**Authors:** Astrid Lehner, Martin Schecklmann, Timm B. Poeppl, Peter M. Kreuzer, Juliette Peytard, Elmar Frank, Berthold Langguth

**Affiliations:** ^1^Department of Psychiatry and Psychotherapy, University of Regensburg, 93053 Regensburg, Germany; ^2^Interdisciplinary Tinnitus Center, University of Regensburg, 93053 Regensburg, Germany

## Abstract

*Background*. Repetitive transcranial magnetic stimulation (rTMS) has shown promising effects in the treatment of chronic subjective tinnitus. However, little is known about maintenance treatment in order to achieve long-lasting improvements. *Objective*. This study addresses the questions whether the repeated application of rTMS treatment can contribute to the maintenance or enhancement of treatment effects and if so in which cases repetitive treatment courses are beneficial. *Methods*. 55 patients with chronic tinnitus were treated with two rTMS treatment courses with ten treatment sessions each. The mean intertreatment interval was 20.65 ± 18.56 months. Tinnitus severity was assessed before and after each treatment course. *Results*. Both treatments were well tolerated and caused significant improvement of tinnitus severity. The main predictor for the outcome of the second treatment was the development of tinnitus distress in the phase between both treatment courses: the more patients worsened in this interval, the more they improved during the second treatment course. *Conclusion*. Repeated application of rTMS seems to be useful in tinnitus management and should preferentially be offered to patients who experience a worsening of their tinnitus during the intertreatment interval, irrespective of their response to the first treatment course.

## 1. Introduction

In the past decade, an increasing number of studies have examined the effectiveness of repetitive transcranial magnetic stimulation (rTMS) as a treatment option for chronic subjective tinnitus (for a review, see [1, 2]). Generally, the reduction of the tinnitus percept and distress after five to ten sessions of rTMS is reported to be mixed with respect to both duration and extent of improvement. While some studies indicate that the improvement vanishes after two weeks [3, 4], other studies observed rather long-lasting effects up to 4 years [5–7]. Likewise, while some patients report no or only little benefit, others improve a lot due to rTMS treatment. Besides this heterogeneous clinical improvement, the actual impact of rTMS treatment on the morphology of the brain seems to be of transient nature [8]. In any case, tinnitus is a chronic condition and the question arises what might be the next therapeutic step either if a patient still feels burdened by his tinnitus after a treatment attempt with rTMS or if the benefit of the treatment wanes over time. In these cases the repeated application of rTMS treatment might be useful. The idea to use repeated stimulation courses to maintain treatment effects is known from electroconvulsive therapy (ECT) of major depression, where immediate treatment effects are of temporary nature as well and where relapses of depressive symptoms are managed successfully by periodic repetition of ECT [9, 10]. Recently, it has even been debated whether rTMS could be used as maintenance method after an initial ECT therapy [11, 12]. Also, there already is some evidence for repeated rTMS treatments from studies examining the benefit of rTMS in patients with psychiatric or neurological disorders (for an overview, see [13]). In patients suffering from depression who had responded to an initial rTMS treatment, both the repetition of the whole treatment schedule after relapse of depressive symptoms [14, 15] and intensive monthly maintenance sessions to prevent the occurrence of a relapse [16, 17] have been shown to be effective. Therefore, it is reasonable to assume that some kind of repeated rTMS treatment might also be useful in tinnitus management. Up to now there is little information on the effect of repeated rTMS therapies in tinnitus patients. Some case reports, small case series, and one study suggest that rTMS “booster” sessions might be effective as maintenance treatment for treatment responders and might even result in more pronounced tinnitus reduction than the initial treatment [13, 18–20]. Furthermore, maintenance treatment has been found to be well tolerated [13]. However, the sample sizes of those studies were small and only initial treatment responders were considered for repeated treatment. It remains unclear whether patients who had not responded to the initial treatment are “rTMS nonresponders” per se or whether they might benefit from rTMS treatment at some other point in time. As the effect of rTMS is known to be state-dependent [21], it is quite conceivable that a nonresponder might benefit from a second treatment course indeed. By presenting data of a large sample of patients who underwent two complete treatment courses of 10 days of rTMS, including both responders and nonresponders to the initial treatment, the current study tries to answer the questions whether (a) repeated courses of rTMS are safe, (b) whether they can contribute to the maintenance of treatment effects or may even enhance treatment response, and (c) in which cases a second treatment course might be beneficial.

## 2. Materials and Methods

Data from 55 patients (43 men, 12 women) with chronic subjective tinnitus were included in the analyses. Inclusion criteria were age over 18 years and chronic subjective tinnitus for at least 6 months. Exclusion criteria were treatable cause of tinnitus and all contraindications for rTMS treatment (pregnancy, epilepsy, cardiac pacemaker, head injury, and metal objects in or around the body which cannot be removed). Demographical data and clinical characteristics of the sample are given in Table 1. All patients underwent two complete treatment courses with each course consisting of ten sessions of rTMS on ten consecutive working days. Treatment was performed at the Tinnitus Center at the University of Regensburg, Germany. All patients provided written informed consent before both treatment courses. The first treatment course was done either in the context of a controlled clinical trial, open-label feasibility studies, or as compassionate use treatment. Part of the data of the first treatment course was therefore already published in the context of the respective study [22–28]. After the first treatment course, patients were informed about the option to repeat rTMS treatment but no appointments were made. This means that the patients decided for themselves if and when they wanted to repeat rTMS treatment. This could be after a worsening of symptoms, because patients hoped for an enhancement of their improvement or because former nonresponders wanted to retry rTMS treatment. The mean interval between both treatment courses was 20.65 ± 18.56 months.

As data was collected over a long period of time (between 2003 and 2014), different treatment protocols were used (see Table 2). Each protocol contained low-frequency (1 Hz) rTMS of the left temporal or temporoparietal cortex with 2000 or 4000 stimuli per day. In the multisite protocols, additional high-frequency stimulation of the left dorsolateral prefrontal (20 Hz) or medial frontal cortex (10 Hz) or low-frequency stimulation of the right temporoparietal cortex (1 Hz) was involved. The high-frequency stimulation was always done first, followed by the low-frequency part. All treatment protocols were used in the context of clinical trials which had been approved by the local ethics committee. Localization of the stimulated areas was either done with a neuronavigational system or by using a standard procedure based on the 10–20 system [29–35]. Stimulation was applied with a Medtronic system with a classical figure-8-coil or, for the medial frontal stimulation, a double-cone-coil (Medtronic, Minneapolis, MN, USA). During treatment, the coil was held by a mechanical arm and the patients were seated comfortably in a reclining chair. Stimulation intensity was set at 110% of the individual resting motor threshold (RMT) for the figure-8-coil and 100% RMT for the double-cone-coil. RMT was defined as the minimal intensity at which motor evoked potentials were 50 *μ*V in amplitude in the right abductor digiti minimi muscle for five out of ten stimulations [36]. Tinnitus distress was assessed before (baseline) and after (day 12) each treatment course using the German version of the tinnitus questionnaire (TQ, [37]).

IBM SPSS Statistics 22 (IBM Corporation, Armonk, NY) was used for data analyses. To test for changes in tinnitus severity due to rTMS treatment, paired *t*-tests were performed for the first and the second treatment course separately. For all further analyses, the difference of the TQ scores between baseline and day 12 was calculated with negative values indicating an improvement in tinnitus severity. Below, those difference scores are named “TQ difference 1” for the first treatment course and “TQ difference 2” for the second treatment course. Furthermore, the TQ difference of the intertreatment interval (ITI) was calculated to represent the development of tinnitus severity in the phase between both treatment courses (“TQ difference ITI”; baseline of the second treatment minus day 12 of the first treatment). To assess which patients benefit from a second treatment course, the following parameters were considered as possible predictors for TQ difference 2: TQ difference 1, the baseline score of the second treatment course, TQ difference ITI, the duration of the intertreatment interval (in months), and the change of treatment protocol from treatment one to treatment two (i.e., if the same protocol was used for both treatment courses, dummy coded). In a first step, all of those parameters were analysed with respect to their relation to TQ difference 2 using product-moment correlations for metric variables and a *t*-test for the discrete variable. All variables with significant influence on TQ difference 2 were entered as regressors into a multiple linear regression analysis (simultaneous model) where TQ difference 2 served as dependent variable. This was done in order to examine which regressors exert most influence if the other regressors are controlled for and to find out whether there are important interaction effects. Finally, in order to find out whether one of the different rTMS protocols was more effective than the others, analyses of variance (ANOVAs) were calculated. The TQ differences of the first or second treatment course were used as dependent variables and the rTMS protocols were used as independent variables (six protocols in the first treatment course, four protocols in the second treatment course; see Table 2). The level of significance was set at 0.05.

## 3. Results

**Figure 1:**
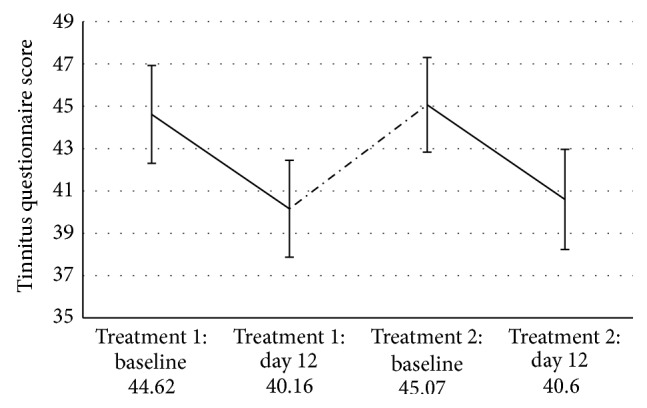
Change of the TQ score over time (mean ± standard error). The intertreatment interval is depicted as dashed line.

**Figure 2:**
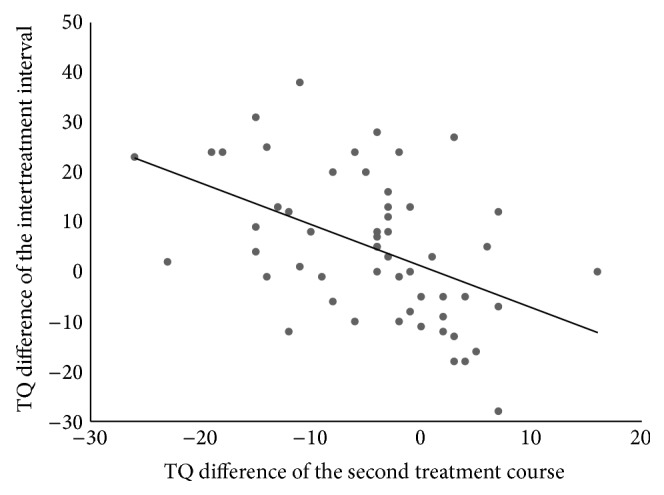
Point diagram showing the relation between the outcome of the second treatment course and the TQ difference of the intertreatment interval.

**Figure 3:**
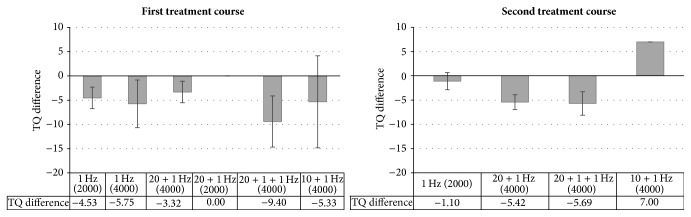
Treatment outcomes (as measured by TQ differences) resulting from the different treatment protocols.

All patients tolerated both treatment courses without any severe adverse effects. All treatments were completed as planned. Paired *t*-tests revealed that both the first and the second treatment course significantly reduced tinnitus severity as measured by the TQ (first treatment course *t*(54) = 3.26, *p* = 0.002; second treatment course *t*(54) = 4.033, *p* < 0.001). Please see Table 1 for mean and standard deviation of the TQ differences.  Figure 1 shows the development of the TQ score over time. The *t*-test which was done to find out whether a change of treatment protocol from treatment one to treatment two had an influence on the outcome of the second treatment revealed no significant effect (*t*(53) = −0.89, *p* = 0.376). The product-moment correlations with TQ difference 2 were not significant for the duration of the intertreatment interval (*r* = −0.167, *p* = 0.223) and the baseline score of the second treatment course (*r* = −0.128, *p* = 0.351). In contrast, the correlations were significant for TQ difference 1 (*r* = 0.282, *p* = 0.037) and TQ difference ITI (*r* = −0.475, *p* < 0.001). Therefore, the latter two variables were entered as regressors in the linear regression analysis. Additionally, an interaction term between both variables was created by multiplying the centred variables. This term was also entered into the regression analysis. The TQ difference of the intertreatment interval significantly predicted the outcome of the second treatment course (*β* = −0.452, *t* = −3.12, and *p* = 0.003) while both TQ difference 1 (*β* = 0.041, *t* = 0.28, and *p* = 0.780) and the interaction term (*β* = −0.013, *t* = −0.11, and *p* = 0.915) were no significant predictors. Thus, TQ difference 1 loses its significant influence on TQ difference 2 if TQ difference ITI is controlled for. The overall model fit was *R* ^2^ = 0.227, *F*(3,51) = 5.00, and *p* = 0.004. The scatter plot in Figure 2 shows the relation between TQ difference 2 and TQ difference ITI. The ANOVAs comparing the TQ differences obtained by the different treatment protocols turned out nonsignificant (*F*(5,49) = 0.37; *p* = 0.869 for the first treatment course; *F*(3,51) = 1.48, *p* = 0.231 for the second treatment course) indicating that none of the protocols was significantly superior (see Figure 3).

## 4. Discussion

This is the first study to examine repeated rTMS treatment courses in a rather large sample of tinnitus patients where responders as well as nonresponders of the initial treatment course were included. Both the first and the second treatment were well tolerated and led to a significant reduction of tinnitus severity. This finding confirms previous findings from smaller samples [13, 18, 19] and further supports the usefulness of repeated rTMS treatment for the management of chronic disorders like tinnitus. As can be seen in Figure 1, the average TQ scores decreased after the first treatment, increased in the intertreatment interval, and improved again during the second treatment course. These group data suggest that the beneficial effect of the first rTMS treatment vanishes over time during the intertreatment interval but can be renewed by repeated rTMS. The regression analysis provides additional insights. It reveals that TQ difference 2 is significantly related to both TQ difference 1 and TQ difference ITI. However, TQ difference 1 loses its significant influence if TQ difference ITI is controlled for. This means that the development of the TQ score during the intertreatment interval is a good predictor for the outcome of the second treatment course and that the outcome of the first treatment course provides hardly any additional predictive information. Consequently, a second treatment attempt might be most promising for patients who worsen between both treatment courses (see Figure 2), irrespective of the success of the first treatment. Interestingly enough, the baseline value of the second treatment is not significantly correlated with TQ difference 2. Thus, patients who worsen between both treatments do not have better outcomes to a second treatment course simply because they have higher baseline values. It was already observed previously that patients whose TQ score had increased in the period before initiation of rTMS benefited more than patients with prior improvement of tinnitus severity [27, 38]. One possible explanation for this effect is that the pretreatment changes in tinnitus severity might reflect a particular neurobiological condition of the brain which makes a response to rTMS more or less likely. As is known from former studies [21, 39, 40] the effect of rTMS depends on the state our brain is in when it is stimulated. Maybe, a worsening of tinnitus is accompanied by a neuronal activation pattern which makes the tinnitus brain more receptible to rTMS than a brain which is currently experiencing no change or improvement of tinnitus severity. This would also explain why the baseline score of the second treatment course, the duration of the intertreatment interval, and the change of the stimulation protocol did not correlate significantly with the outcome of the second treatment course—the baseline score alone tells us little about the process the brain is currently in—nor does the duration of the intertreatment interval. And a change of the stimulation protocol might only be promising if our brain is in a state susceptible to rTMS intervention and if the new protocol fits this state. This is only speculation, of course, and future controlled studies are needed to shed more light on the relation between the activational state of the tinnitus brain and its responsiveness to rTMS and to find out whether there are clinical markers (like the TQ difference of the intertreatment interval) which reflect such an advantageous state reliably.

With respect to the repetition of rTMS treatment in general, the current study clearly shows that the second treatment course reduces tinnitus severity just as well as the first one. This is in line with former papers which also reported good response to maintenance treatment [13, 18, 19, 41]. As there are many possibilities as to when, how, and to whom repeated treatments can be offered, it is not surprising that past studies and case reports differ a lot regarding the strategy used for repeated rTMS treatments. In the current study, we decided to make as little specification of those variables as possible. This was done in order to be able to get an overall impression of repeated courses of rTMS for tinnitus patients and to find out which variables are of more or less importance for future studies. The only parameter that was determined in this study was the number of sessions: patients were treated with the full treatment course of ten sessions of rTMS. No fixed time schedule was used though but patients were retreated whenever they requested it. This design has the advantage that it reflects the typical clinical situation in which a patient presents for a repeated rTMS treatment. The correlation between the duration of the intertreatment interval and the outcome of the second treatment course was not significant, indicating that it might be not decisive for treatment response if repeated treatments are applied within few months after the first treatment attempt or after years.

Similar to the time schedule, the sample of patients for this study was also not determined beforehand. All previous studies only considered initial treatment responders for repeated rTMS courses, preventing the investigation of the question if the retreatment of a nonresponder is adequate. The present results show that also nonresponders might benefit from further rTMS treatment and that the response to the initial treatment is a good but not a sufficient predictor for the outcome of the second treatment course. Particularly if nonresponders experience a deterioration of their tinnitus, another treatment attempt might be reasonable.

It turned out that the treatment protocols did not differ significantly with respect to treatment outcome neither in the first nor in the second treatment course. This is in line with past studies indicating that a superiority of combined protocols as compared with single-site protocols is not present immediately after rTMS treatment but only after a certain follow-up period (e.g., [22, 28]). As, in the current study, treatment outcome was only assessed directly after the last treatment session but not after a follow-up period, a difference between protocols was not to be expected.

The explorative character of our study entails limitations on the conclusions which can be drawn. Neither subjects nor experimenters were blinded regarding the second treatment course and the study lacks a wait list or placebo control group. Furthermore, there was a self-selection bias of patients who underwent the second rTMS treatment, and also the interval between the two treatment courses was not standardized. Therefore it cannot be exactly determined whether the observed effects are entirely rTMS specific and to which extent unspecific effects like a tendency to the mean may have contributed. Furthermore, the protocols used for rTMS treatment were not kept constant: six different protocols were used for the first treatment course and four different protocols for the second treatment course. Nevertheless, the results provide valuable information for future controlled trials which are clearly needed to investigate maintenance rTMS in more detail. In summary, this study demonstrated that (a) repeated courses of rTMS treatment cause a significant change of tinnitus severity and (b) the change of tinnitus severity during the intertreatment interval is a good predictor for treatment outcome of the second treatment course with patients whose tinnitus worsens during this interval benefitting most from the second treatment. It is particularly important to note that this is also true for initial nonresponders. If a further deterioration of their tinnitus happens, a repetition of rTMS treatment might definitely be reasonable in those patients.

## 5. Conclusions

Presenting a large sample of patients with chronic subjective tinnitus who were treated with two full courses of rTMS treatment, the current study shows that the repeated application of rTMS is well tolerated and represents a useful tool in tinnitus management. A second treatment attempt is especially promising for patients who had experienced a worsening of their tinnitus during the intertreatment interval. It is important to note that this relation is also true for patients who did not respond to the first treatment course: if those patients present with a deterioration of symptoms, they might benefit from a second treatment course indeed.

## Figures and Tables

**Table 1:** Demographical data and clinical characteristics of the sample.

Gender	43 males, 12 females
Age (years)	52.49 ± 11.42
Tinnitus duration (years)^*^	7.94 ± 7.15
Tinnitus laterality	5 right
	8 left
	12 both ears worse left
	8 both ears worse right
	19 both ears equally
	3 inside the head
Intertreatment interval (months)	20.65 ± 18.56
Identical treatment protocol for both treatment courses	30 yes, 25 no
TQ difference 1	−4.45 ± 10.13
TQ difference 2	−4.47 ± 8.23
TQ difference intertreatment interval	4.91 ± 14.42

Data are given as mean ± standard deviation.

^*^Before starting of first treatment course.

**Table 2:** Treatment protocols used in the first and second treatment courses.

	Number of patients treated	
	1st treatment	2nd treatment
Left temporal rTMS, 1 Hz, 2000 stimuli/day	19	10
Left temporal rTMS, 1 Hz, 4000 stimuli/day	4	—
Left temporal (1 Hz) plus left frontal (20 Hz) rTMS, 2000 stimuli/day	2	—
Left temporal (1 Hz) plus left frontal (20 Hz) rTMS, 4000 stimuli/day	22	31
Left and right temporoparietal (1 Hz) plus left frontal (20 Hz) rTMS, 4000 stimuli/day	5	13
Left temporoparietal (1 Hz) plus medial frontal (10 Hz), 4000 stimuli/day	3	1
